# Supplementary material for: Assessment of oxidative stress in autism spectrum disorder using reactive oxygen metabolites and biological antioxidant potential
Source: PLoS One. 2020 May 22;15(5):e0233550. doi: 10.1371/journal.pone.0233550 (PMC7244111; doi:10.1371/journal.pone.0233550)
Supplement: S1 Table — (DOCX) [file pone.0233550.s001.docx]

【TD】

| **Age** | **Sex** | **d-ROMs** | **BAP** | **d-ROMs/BAP** | **BAP/d-ROMs** | **PARS** |
| --- | --- | --- | --- | --- | --- | --- |
| 2 | F | 498 | 2298 | 0.21671018 | 4.61445783 | 6 |
| 3 | F | 358 | 2051 | 0.174549 | 5.72905028 | 2 |
| 3 | F | 422 | 2564 | 0.16458658 | 6.07582938 | 1 |
| 3 | M | 428 | 1795 | 0.23844011 | 4.19392523 | 2 |
| 3 | M | 493 | 1745 | 0.28252149 | 3.53955375 | 1 |
| 3 | M | 520 | 2109 | 0.24656235 | 4.05576923 | 5 |
| 3 | M | 415 | 1955 | 0.21227621 | 4.71084337 | 2 |
| 4 | F | 485 | 2011 | 0.24117355 | 4.14639175 | 2 |
| 4 | F | 478 | 2598 | 0.18398768 | 5.43514644 | 0 |
| 4 | F | 384 | 2109 | 0.18207681 | 5.4921875 | 3 |
| 4 | M | 451 | 1890 | 0.23862434 | 4.19068736 | 0 |
| 4 | M | 397 | 2288 | 0.17351399 | 5.76322418 | 1 |
| 4 | M | 421 | 2005 | 0.20997506 | 4.76247031 | 3 |
| 5 | F | 458 | 2657 | 0.17237486 | 5.80131004 | 3 |
| 5 | F | 415 | 2205 | 0.18820862 | 5.31325301 | 1 |
| 5 | F | 395 | 2291 | 0.17241379 | 5.8 | 0 |
| 5 | F | 325 | 2198 | 0.14786169 | 6.76307692 | 1 |
| 5 | M | 396 | 2504 | 0.15814696 | 6.32323232 | 2 |
| 5 | M | 345 | 1953 | 0.17665131 | 5.66086957 | 1 |
| 5 | M | 352 | 2493 | 0.14119535 | 7.08238636 | 0 |
| 5 | M | 396 | 1600 | 0.2475 | 4.04040404 | 1 |
| 6 | F | 334 | 1974 | 0.16919959 | 5.91017964 | 2 |
| 6 | F | 457 | 2330 | 0.19613734 | 5.09846827 | 0 |
| 6 | F | 314 | 2529 | 0.12415975 | 8.05414013 | 1 |
| 6 | M | 332 | 2108 | 0.15749526 | 6.34939759 | 2 |
| 6 | M | 348 | 2490 | 0.13975904 | 7.15517241 | 1 |
| 6 | M | 404 | 2670 | 0.15131086 | 6.60891089 | 0 |
| 6 | M | 323 | 2371 | 0.13622944 | 7.34055728 | 3 |
| 7 | F | 335 | 2270 | 0.14757709 | 6.7761194 | 2 |
| 7 | F | 312 | 2432 | 0.12828947 | 7.79487179 | 2 |
| 7 | F | 331 | 2313 | 0.14310419 | 6.98791541 | 2 |
| 7 | M | 312 | 2149 | 0.14518381 | 6.88782051 | 3 |
| 7 | M | 289 | 2047 | 0.14118222 | 7.08304498 | 0 |
| 7 | M | 352 | 2865 | 0.12286213 | 8.13920455 | 2 |
| 7 | M | 361 | 2448 | 0.14746732 | 6.78116343 | 5 |
| 8 | F | 293 | 2281 | 0.12845243 | 7.78498294 | 2 |
| 8 | F | 356 | 2401 | 0.14827155 | 6.74438202 | 0 |
| 8 | M | 315 | 2380 | 0.13235294 | 7.55555556 | 0 |
| 8 | M | 321 | 2366 | 0.13567202 | 7.37071651 | 1 |
| 8 | M | 320 | 2653 | 0.12061817 | 8.290625 | 1 |
| 9 | F | 310 | 2474 | 0.12530315 | 7.98064516 | 1 |
| 9 | F | 265 | 2571 | 0.10307273 | 9.70188679 | 1 |
| 9 | M | 290 | 1840 | 0.1576087 | 6.34482759 | 5 |
| 9 | M | 281 | 2620 | 0.10725191 | 9.32384342 | 2 |
| 9 | M | 307 | 2791 | 0.10999642 | 9.09120521 | 1 |
| 9 | M | 333 | 2550 | 0.13058824 | 7.65765766 | 1 |
| 10 | F | 301 | 2095 | 0.14367542 | 6.96013289 | 1 |
| 10 | F | 273 | 2497 | 0.1093312 | 9.14652015 | 1 |
| 10 | F | 310 | 2326 | 0.13327601 | 7.50322581 | 0 |
| 10 | F | 283 | 2474 | 0.11438965 | 8.74204947 | 4 |
| 10 | M | 270 | 1995 | 0.13533835 | 7.38888889 | 4 |
| 10 | M | 297 | 2981 | 0.099631 | 10.037037 | 1 |
| 11 | F | 260 | 2014 | 0.12909633 | 7.74615385 | 3 |
| 11 | F | 278 | 2166 | 0.12834718 | 7.79136691 | 0 |
| 11 | M | 298 | 2145 | 0.13892774 | 7.19798658 | 0 |
| 11 | M | 278 | 2821 | 0.09854661 | 10.147482 | 1 |
| 12 | F | 292 | 2373 | 0.12305099 | 8.12671233 | 0 |
| 12 | F | 301 | 2201 | 0.13675602 | 7.31229236 | 1 |
| 12 | F | 309 | 2500 | 0.1236 | 8.09061489 | 1 |
| 12 | M | 260 | 2482 | 0.10475423 | 9.54615385 | 3 |
| 12 | M | 297 | 2294 | 0.12946818 | 7.72390572 | 0 |
| 12 | M | 254 | 2301 | 0.11038679 | 9.05905512 | 3 |
| 13 | F | 252 | 2022 | 0.12462908 | 8.02380952 | 2 |
| 13 | F | 307 | 2512 | 0.12221338 | 8.18241042 | 0 |
| 13 | F | 284 | 2802 | 0.10135617 | 9.86619718 | 4 |
| 13 | M | 299 | 2311 | 0.12938122 | 7.72909699 | 0 |
| 13 | M | 268 | 2115 | 0.12671395 | 7.89179104 | 0 |
| 14 | F | 282 | 2105 | 0.13396675 | 7.46453901 | 6 |
| 14 | F | 308 | 2261 | 0.13622291 | 7.34090909 | 0 |
| 14 | F | 314 | 2044 | 0.15362035 | 6.50955414 | 0 |
| 14 | M | 292 | 2613 | 0.11174895 | 8.94863014 | 2 |
| 14 | M | 255 | 2352 | 0.10841837 | 9.22352941 | 1 |
| 14 | M | 296 | 2401 | 0.12328197 | 8.11148649 | 1 |
| 15 | F | 257 | 2172 | 0.11832413 | 8.45136187 | 0 |
| 15 | F | 265 | 2575 | 0.10291262 | 9.71698113 | 2 |
| 15 | F | 255 | 2206 | 0.11559383 | 8.65098039 | 0 |
| 15 | M | 278 | 2011 | 0.13823968 | 7.23381295 | 0 |

【ASD】

| **Age** | **Sex** | **d-ROMs** | **BAP** | **d-ROM/BAP** | **BAP/d-ROM** | **PARS** | **IQ** | **Method** | **Pyruvate** | **Pyruvate** | **L/P ratio** |
| --- | --- | --- | --- | --- | --- | --- | --- | --- | --- | --- | --- |
| 2 | M | 420 | 2774 | 0.15140591 | 6.6047619 | 13 | 63 | K式 | 16 | 1.21 | 13.22314 |
| 2 | F | 543 | 3105 | 0.17487923 | 5.71823204 | 21 | 83 | 遠城寺 | 9.7 | 0.91 | 10.65934 |
| 3 | M | 400 | 1967 | 0.20335536 | 4.9175 | 29 | 76 | K式 | 5.5 | 0.54 | 10.18519 |
| 3 | M | 331 | 2169 | 0.15260489 | 6.55287009 | 22 | 77 | DQ | 4.9 | 0.36 | 13.61111 |
| 3 | M | 512 | 3020 | 0.16953642 | 5.8984375 | 35 | 107 | WISC-Ⅳ | 11.9 | 0.95 | 12.52632 |
| 4 | M | 435 | 3106 | 0.14005151 | 7.14022989 | 13 | 68 | K式 | 13.7 | 0.99 | 13.83838 |
| 4 | F | 385 | 1707 | 0.22554189 | 4.43376623 | 38 | 70 | 遠城寺 | 8.9 | 0.72 | 12.36111 |
| 4 | M | 434 | 2701 | 0.16068123 | 6.2235023 | 14 | 72 | KABCⅡ | 14.2 | 1.13 | 12.56637 |
| 4 | M | 565 | 2744 | 0.20590379 | 4.85663717 | 33 | 81 | WISC-Ⅳ | 6.1 | 0.51 | 11.96078 |
| 4 | M | 455 | 2270 | 0.20044053 | 4.98901099 | 16 | 85 | WISC-Ⅳ | 7.5 | 0.68 | 11.02941 |
| 4 | M | 505 | 2751 | 0.18356961 | 5.44752475 | 37 | 91 | KABCⅡ | 10.3 | 0.91 | 11.31868 |
| 4 | M | 411 | 3660 | 0.11229508 | 8.90510949 | 15 | 92 | K式 | 7.2 | 0.48 | 15 |
| 4 | M | 441 | 3409 | 0.12936345 | 7.73015873 | 14 | 92 | WISC-Ⅳ | 7.8 | 0.67 | 11.64179 |
| 4 | M | 383 | 3018 | 0.12690524 | 7.87989556 | 17 | 102 | WISC-Ⅳ | 17.5 | 0.94 | 18.61702 |
| 4 | M | 422 | 2847 | 0.1482262 | 6.7464455 | 15 | 111 | WPPS1 | 13.5 | 0.78 | 17.30769 |
| 5 | M | 536 | 3509 | 0.15275007 | 6.54664179 | 11 | 50 | KABCⅡ | 8.9 | 0.64 | 13.90625 |
| 5 | F | 426 | 2454 | 0.17359413 | 5.76056338 | 35 | 54 | K式 | 6.9 | 0.55 | 12.54545 |
| 5 | M | 420 | 3391 | 0.12385727 | 8.07380952 | 12 | 58 | WISC-Ⅳ | 19.5 | 1.22 | 15.98361 |
| 5 | M | 403 | 2455 | 0.16415479 | 6.09181141 | 16 | 63 | WISC-Ⅳ | 15.5 | 0.78 | 19.87179 |
| 5 | F | 336 | 2449 | 0.13719886 | 7.28869048 | 19 | 66 | K式 | 5.4 | 0.52 | 10.38462 |
| 5 | M | 563 | 2985 | 0.18860972 | 5.30195382 | 14 | 67 | KABCⅡ | 27.8 | 1.81 | 15.35912 |
| 5 | M | 294 | 1674 | 0.17562724 | 5.69387755 | 15 | 71 | WISC-Ⅳ | 13.1 | 0.97 | 13.50515 |
| 5 | M | 485 | 2831 | 0.17131756 | 5.8371134 | 16 | 74 | WISC-Ⅳ | 17.5 | 1.14 | 15.35088 |
| 5 | F | 463 | 3235 | 0.1431221 | 6.98704104 | 10 | 78 | WISC-Ⅳ | 14.2 | 1.22 | 11.63934 |
| 5 | M | 435 | 2504 | 0.17372204 | 5.75632184 | 16 | 80 | WISC-Ⅳ | 12.9 | 0.68 | 18.97059 |
| 5 | F | 370 | 2501 | 0.14794082 | 6.75945946 | 12 | 83 | WISC-Ⅳ | 7.6 | 0.76 | 10 |
| 5 | M | 508 | 1970 | 0.25786802 | 3.87795276 | 34 | 90 | WISC-Ⅳ | 9.7 | 0.77 | 12.5974 |
| 5 | M | 365 | 2912 | 0.12534341 | 7.97808219 | 9 | 91 | WISC-Ⅳ | 15.8 | 0.86 | 18.37209 |
| 5 | M | 356 | 2542 | 0.14004721 | 7.14044944 | 13 | 91 | WISC-Ⅳ | 12.2 | 0.83 | 14.6988 |
| 5 | M | 386 | 2768 | 0.13945087 | 7.17098446 | 20 | 92 | WISC-Ⅳ | 12.8 | 0.7 | 18.28571 |
| 5 | F | 401 | 2655 | 0.15103578 | 6.62094763 | 18 | 93 | WISC-Ⅳ | 14.6 | 1.03 | 14.17476 |
| 5 | M | 275 | 1670 | 0.16467066 | 6.07272727 | 10 | 93 | WISC-Ⅳ | 9.7 | 0.84 | 11.54762 |
| 5 | M | 518 | 2496 | 0.20753205 | 4.81853282 | 11 | 94 | WISC-Ⅳ | 11.6 | 0.96 | 12.08333 |
| 5 | F | 422 | 2706 | 0.15594974 | 6.41232227 | 13 | 101 | WISC-Ⅳ | 16.6 | 1.82 | 9.120879 |
| 5 | F | 391 | 2765 | 0.14141049 | 7.07161125 | 10 | 106 | WISC-Ⅳ | 7.7 | 0.57 | 13.50877 |
| 5 | F | 455 | 2915 | 0.15608919 | 6.40659341 | 24 | 106 | WISC-Ⅳ | 16.3 | 1.26 | 12.93651 |
| 5 | M | 561 | 2940 | 0.19081633 | 5.24064171 | 16 | 107 | WISC-Ⅳ | 8.9 | 0.58 | 15.34483 |
| 5 | M | 411 | 2596 | 0.15832049 | 6.3163017 | 15 | 111 | WISC-Ⅳ | 7.4 | 0.53 | 13.96226 |
| 6 | F | 446 | 2508 | 0.17783094 | 5.62331839 | 18 | 69 | WISC-Ⅳ | 8.3 | 0.59 | 14.0678 |
| 6 | M | 410 | 2721 | 0.1506799 | 6.63658537 | 8 | 75 | WISC-Ⅳ | 11 | 0.92 | 11.95652 |
| 6 | M | 410 | 3298 | 0.12431777 | 8.04390244 | 15 | 79 | WISC-Ⅳ | 19.7 | 1.32 | 14.92424 |
| 6 | M | 431 | 3658 | 0.11782395 | 8.48723898 | 15 | 82 | KABC | 16.6 | 1.2 | 13.83333 |
| 6 | F | 433 | 2335 | 0.18543897 | 5.3926097 | 13 | 83 | WISC-Ⅳ | 10.5 | 0.95 | 11.05263 |
| 6 | M | 501 | 2865 | 0.17486911 | 5.71856287 | 17 | 83 | WISC-Ⅳ | 12.1 | 1.07 | 11.30841 |
| 6 | M | 391 | 2719 | 0.14380287 | 6.95396419 | 12 | 88 | WISC-Ⅳ | 7.3 | 0.85 | 8.588235 |
| 6 | M | 554 | 3101 | 0.17865205 | 5.59747292 | 14 | 90 | WISC-Ⅳ | 11.5 | 0.83 | 13.85542 |
| 6 | M | 425 | 3101 | 0.13705256 | 7.29647059 | 14 | 96 | KABC | 17.4 | 1.37 | 12.70073 |
| 6 | M | 427 | 2442 | 0.17485667 | 5.71896956 | 16 | 103 | WISC-Ⅲ | 10 | 0.81 | 12.34568 |
| 6 | M | 327 | 2231 | 0.14657104 | 6.82262997 | 7 | 109 | WISC-Ⅳ | 10.3 | 0.62 | 16.6129 |
| 6 | F | 480 | 2555 | 0.18786693 | 5.32291667 | 13 | 123 | WISC-Ⅳ | 5.2 | 0.5 | 10.4 |
| 7 | M | 325 | 2208 | 0.14719203 | 6.79384615 | 21 | 71 | WISC-Ⅳ | 10.6 | 0.76 | 13.94737 |
| 7 | M | 277 | 1874 | 0.14781217 | 6.76534296 | 19 | 77 | WISC-Ⅳ | 4.5 | 0.42 | 10.71429 |
| 7 | M | 593 | 2683 | 0.22102124 | 4.52445194 | 16 | 80 | WISC-Ⅳ | 6.5 | 0.56 | 11.60714 |
| 7 | M | 421 | 2758 | 0.15264685 | 6.55106888 | 17 | 80 | WISC-Ⅳ | 9.8 | 0.77 | 12.72727 |
| 7 | M | 488 | 2925 | 0.16683761 | 5.99385246 | 22 | 82 | KABCⅡ | 7.6 | 0.57 | 13.33333 |
| 7 | M | 452 | 2815 | 0.16056838 | 6.22787611 | 15 | 83 | WISC-Ⅳ | 9.7 | 0.91 | 10.65934 |
| 7 | F | 447 | 3236 | 0.1381335 | 7.2393736 | 17 | 87 | WISC-Ⅳ | 11.4 | 0.91 | 12.52747 |
| 7 | M | 444 | 2851 | 0.15573483 | 6.42117117 | 20 | 90 | WISC-Ⅳ | 9 | 0.84 | 10.71429 |
| 7 | F | 353 | 1976 | 0.17864372 | 5.59773371 | 36 | 98 | WISC-Ⅳ | 32.6 | 1 | 32.6 |
| 7 | M | 509 | 1799 | 0.28293496 | 3.53438114 | 13 | 103 | KABCⅡ | 6.5 | 0.48 | 13.54167 |
| 8 | M | 440 | 2171 | 0.20267158 | 4.93409091 | 23 | 49 | WISC-Ⅲ | 11.8 | 0.84 | 14.04762 |
| 8 | F | 323 | 2767 | 0.11673292 | 8.56656347 | 16 | 77 | WISC-Ⅳ | 7.9 | 0.54 | 14.62963 |
| 8 | F | 442 | 2621 | 0.16863792 | 5.92986425 | 19 | 79 | WISC-Ⅳ | 6 | 0.53 | 11.32075 |
| 8 | M | 408 | 2875 | 0.14191304 | 7.04656863 | 17 | 84 | WISC-Ⅳ | 16.3 | 1.04 | 15.67308 |
| 8 | M | 389 | 1923 | 0.20228809 | 4.94344473 | 19 | 88 | WISC-Ⅳ | 31.4 | 1.68 | 18.69048 |
| 8 | M | 452 | 2810 | 0.16085409 | 6.21681416 | 19 | 94 | WISC-Ⅳ | 12.2 | 0.82 | 14.87805 |
| 8 | M | 385 | 2121 | 0.18151815 | 5.50909091 | 17 | 94 | WISC-Ⅳ | 8.6 | 0.57 | 15.08772 |
| 8 | M | 296 | 2314 | 0.12791703 | 7.81756757 | 14 | 100 | WISC-Ⅳ | 17.1 | 1.02 | 16.76471 |
| 8 | M | 374 | 2529 | 0.14788454 | 6.76203209 | 19 | 101 | WISC-Ⅳ | 11.8 | 0.88 | 13.40909 |
| 9 | M | 412 | 2288 | 0.18006993 | 5.55339806 | 23 | 58 | WISC-Ⅳ | 6.6 | 0.59 | 11.18644 |
| 9 | M | 296 | 2372 | 0.12478921 | 8.01351351 | 23 | 65 | WISC-Ⅳ | 13.2 | 0.79 | 16.70886 |
| 9 | F | 359 | 3122 | 0.11499039 | 8.69637883 | 15 | 72 | WISC-Ⅳ | 12.3 | 0.79 | 15.56962 |
| 9 | F | 427 | 2459 | 0.17364782 | 5.7587822 | 24 | 75 | WISC-Ⅳ | 8.7 | 0.74 | 11.75676 |
| 9 | M | 477 | 2642 | 0.18054504 | 5.53878407 | 19 | 88 | WISC-Ⅳ | 10.5 | 0.64 | 16.40625 |
| 9 | M | 499 | 2763 | 0.1806008 | 5.53707415 | 15 | 90 | WISC-Ⅳ | 4.7 | 0.53 | 8.867925 |
| 9 | F | 398 | 2560 | 0.15546875 | 6.4321608 | 20 | 97 | WISC-Ⅳ | 8.1 | 0.71 | 11.40845 |
| 9 | M | 420 | 2605 | 0.16122841 | 6.20238095 | 21 | 113 | WISC-Ⅳ | 9.3 | 0.86 | 10.81395 |
| 9 | F | 449 | 2005 | 0.22394015 | 4.46547884 | 23 | 123 | WISC-Ⅲ | 8.1 | 0.65 | 12.46154 |
| 10 | M | 491 | 2952 | 0.16632791 | 6.01221996 | 22 | 75 | WISC-Ⅳ | 9.8 | 0.79 | 12.40506 |
| 10 | M | 395 | 3155 | 0.1251981 | 7.98734177 | 17 | 84 | WISC-Ⅳ | 7.4 | 0.61 | 12.13115 |
| 10 | F | 384 | 1914 | 0.20062696 | 4.984375 | 24 | 85 | WISC-Ⅳ | 6.6 | 0.53 | 12.45283 |
| 10 | M | 322 | 3042 | 0.10585141 | 9.44720497 | 13 | 95 | WISC-Ⅳ | 17 | 1.51 | 11.25828 |
| 10 | M | 441 | 2788 | 0.15817791 | 6.32199546 | 26 | 98 | WISC-Ⅳ | 9.5 | 0.77 | 12.33766 |
| 10 | F | 396 | 2501 | 0.15833667 | 6.31565657 | 23 | 101 | WISC-Ⅳ | 6.1 | 0.49 | 12.44898 |
| 10 | M | 474 | 2682 | 0.17673378 | 5.65822785 | 21 | 103 | WISC-Ⅳ | 15.7 | 1.28 | 12.26563 |
| 10 | M | 503 | 3227 | 0.15587233 | 6.41550696 | 18 | 131 | WISC-Ⅳ | 12.3 | 0.9 | 13.66667 |
| 11 | M | 358 | 2346 | 0.15260017 | 6.55307263 | 15 | 84 | WISC-Ⅳ | 10.4 | 0.81 | 12.83951 |
| 11 | M | 447 | 2441 | 0.18312167 | 5.46085011 | 25 | 85 | WISC-Ⅳ | 6.5 | 0.67 | 9.701493 |
| 11 | M | 371 | 2078 | 0.17853705 | 5.60107817 | 28 | 102 | WISC-Ⅳ | 7.1 | 0.65 | 10.92308 |
| 12 | M | 412 | 2612 | 0.15773354 | 6.33980583 | 19 | 60 | WISC-Ⅳ | 6.4 | 0.6 | 10.66667 |
| 12 | F | 354 | 2749 | 0.1287741 | 7.76553672 | 17 | 81 | WISC-Ⅳ | 8.1 | 0.73 | 11.09589 |
| 12 | M | 483 | 2851 | 0.16941424 | 5.90269151 | 11 | 95 | WISC-Ⅳ | 16.2 | 0.93 | 17.41935 |
| 12 | M | 320 | 2888 | 0.11080332 | 9.025 | 28 | 100 | WISC-Ⅳ | 11.7 | 1.01 | 11.58416 |
| 13 | M | 388 | 2526 | 0.15360253 | 6.51030928 | 11 | 104 | WISC-Ⅳ | 9.8 | 0.78 | 12.5641 |
| 14 | M | 471 | 2701 | 0.17437986 | 5.73460722 | 30 | 83 | WISC-Ⅲ | 11.3 | 0.86 | 13.13953 |
| 14 | M | 377 | 1752 | 0.21518265 | 4.64721485 | 34 | 98 | WISC-Ⅳ | 8.9 | 0.63 | 14.12698 |
| 14 | M | 451 | 2617 | 0.17233473 | 5.80266075 | 15 | 103 | WISC-Ⅳ | 10.9 | 0.7 | 15.57143 |
| 15 | F | 389 | 2824 | 0.13774788 | 7.2596401 | 25 | 84 | WISC-Ⅳ | 14.7 | 1 | 14.7 |
