# Supplementary material for: Assessment of oxidative stress in autism spectrum disorder using reactive oxygen metabolites and biological antioxidant potential
Source: PLoS One. 2020 May 22;15(5):e0233550. doi: 10.1371/journal.pone.0233550 (PMC7244111; doi:10.1371/journal.pone.0233550)
Supplement: Attachment — Submitted filename: Response to editor comments.docx [file pone.0233550.s002.docx]

3 February, 2020

Dear Dr. Ece Uzun, PhD (Editor)

Thank you for inviting us to submit a revised draft of our manuscript entitled, “Assessment of Oxidative Stress in Autism Spectrum Disorder Using Reactive Oxygen Metabolites and Biological Antioxidant Potential” to PLOS ONE. We also appreciate the time and effort you and each of the reviewers have dedicated to providing insightful feedback on ways to strengthen our paper. Thus, it is with great pleasure that we resubmit our article for further consideration. We have incorporated changes that reflect the detailed suggestions you have graciously provided. We also hope that our edits and the responses we provide below satisfactorily address all the issues and concerns you and the reviewers have noted.

Sincerely yours,

Masahito Morimoto

Corresponding author

Our response to the editor comment is as follow:

**Comment 1:**

Line 44 You talk about secondary symptoms in autism, but these are co-morbidities and a certain percentage of patients have those. Please clarify this and add what percentage of patients have insomnia, depression and anxiety.

**Response:**

**We wish to thank the Editor for this comment.**

As pointed out, the percentage of comorbidities (insomnia, depression and anxiety) of the subjects in this study was added to "Subjects" in "Methods". As comorbidities, insomnia 20(20.4%), depression 8 (8.2%) and anxiety 37 (37.8%) were observed.

**Comment 2:**

Line 49: Please reference the last sentence in paragraph 1.

**Response:**

Yes, we added references.

**Comment 3:**

Line 57: Please explain in detail why the markers can not be measured rapidly? Please clarify what you mean from rapidly? How long does it take to run the test and what is the benefit of measuring it fast vs slow?

**Response:**

We wish to thank the Editor for this comment.

As with other illnesses, it is beneficial for patients that tests that affect the assessment of ASD are quick and simple. Because both d-ROMs and BAP can be measured in the examination room in about 5 minutes each, we speculated that they may be superior to other markers. NOS, GST, PON-1, etc., require the use of more complex testing equipment than d-ROMs / BAP measuring equipment in the laboratory.

We have revised the text for these contents.

**Comment 4:**

Line 63: Please explain the biological relevance of d-ROMS and BAP in autism.

**Response:**

We appreciate the Editor's comment on this point.

We added the biological relationship between d-ROMs and BAP in ASD as follows.

There has been no report on the relationship between ASD and d-ROMs test.

However, the d-ROMs test is a method in which serum hydroperoxides generates alkoxy radicals and peroxy radicals by the Fenton reaction with iron ions, and quantitatively measures them. There are several reports on the relationship between oxidative stress evaluation by ASD and Fenton reaction, and d-ROMs seems to be a method applicable to oxidative stress evaluation as well.

The BAP test is a test for examining the ability to reduce Fe3^+^ to Fe2^+^. There are reports investigating the relationship between inflammation markers and iron reduction in ASD, suggesting that it can be applied to the evaluation of oxidative stress in ASD.

**Comment 5:**

Methods: Is "Measurement items" a sub-title? Please exclude this as this section is part of methods. Please follow the author guidelines fro proper sub-section titles.

**Response:**

In accordance with the author guidelines, "Measurement items" of "Methods" have been excluded.

**Comment 6:**

Line 92: What does ESR stand for?

**Response:**

ESR stands for Electron Spin Resonance. Contents have been added.

**Comment 7:**

Figures: Please exclude "This the Fig 1 Title" or "This is the Fig 1 legend" statements from all figures. Please follow the author guidelines for proper figure title, legend and captions. Please explain the results observed in a specific figure in detail in the figure captions.

**Response:**

As you pointed out, the figure title and legend have been revised accordance with the author guidelines. In addition, figure captions have been added.

**Comment 8:**

Results: First paragraph should be moved to Methods section as it is describing the cohort. I would recommend to start the results section with a brief introduction of what was done in the study following with the description of results.

**Response:**

We appreciate the Editor's comment on this point.

As you pointed out, the contents of Table 1 are not about "Results" but about "Methods" subjects. Therefore, the content of Table 1 has been moved to the "Methods" section.

Again, thank you for giving us the opportunity to strengthen our manuscript with your valuable comments and queries. We have worked hard to incorporate your feedback and hope that these revisions persuade you to accept our submission.

Masahito Morimoto (Corresponding Author)

Japanese Red Cross Tokushima Hinomine Rehabilitation Center for People with Disabilities

Address: 4-1Shinbiraki, chuden-cho, Komathushima-shi, Tokushima, 773-0015 Japan

Phone : +81-885-32-0903	Fax : +81-885-33-3037

E-mail address: [morimoto@hinomine-mrc.jp](mailto:morimoto@hinomine-mrc.jp)
