# Supplementary material for: Assessment of oxidative stress in autism spectrum disorder using reactive oxygen metabolites and biological antioxidant potential
Source: PLoS One. 2020 May 22;15(5):e0233550. doi: 10.1371/journal.pone.0233550 (PMC7244111; doi:10.1371/journal.pone.0233550)
Supplement: Attachment — Submitted filename: Response to reviewers.docx [file pone.0233550.s003.docx]

April 17, 2020

Dear Reviewer #2

We thank the reviewer for fruitful suggestions, especially for suggesting the better terms and sentences. We have revised the manuscript PONE-D-19-26530R1 “Assessment of oxidative stress in autism spectrum disorder using reactive oxygen metabolites and biological antioxidant potential” on the basis of the reviewer's comments.

We look forward to a publication of our manuscript in PLOS ONE.

Sincerely yours,

Masahito Morimoto

Corresponding author

Our response to the reviewer comment is as follow:

**Comment 1:**

In the introduction-line 61 -wording is clumsy, change‘that tests that’ to ‘that tests which’- less redundant.

**Response:**

We corrected as you pointed out.

**Comment 2:**

Also, change ‘illnesses’ to ‘conditions’- as ASD is not really an ‘illness’.

**Response:**

We corrected as you pointed out.

**Comment 3:**

Also be consistent on capitalization for ‘Fenton’.

**Response:**

We become consistent on capitalization for ‘Fenton’.

**Comment 4:**

Wording is bad on lined 103-104 ‘Analysis results were 2-6 years p = 0.276, 7-15 years p = 0.425.

**Response:**

We deleted the sentence ‘Analysis results were 2-6 years p = 0.276, 7-15 years p = 0.425.’, because it was irrelevant to the explanation in Table 1.

**Comment 5:**

ASD measured lactate and pyruvate’ I don’t know what the first sentence means at all, and how did ‘ASD’ measure lactate and pyruvate? Do the authors mean ‘Lactate and pyruvate was measured in ASD participants’? Please clarify.

**Response:**

We appreciate the Reviewer's comment on this point.

I changed the text as follows.

`Lactate and pyruvate was measured in ASD participants, and calculated the lactate/pyruvate ratio. As a result, no patients had a ratio>20, and there were no symptoms or complications suspected of mitochondrial disease.'

And since it is not related to Table 1, we wrote it in another paragraph.

**Comment 6:**

Line 134- change ‘generates’ to ‘is generated’.

**Response:**

We corrected as you pointed out.

**Comment 7:**

Line 138- change ‘has a report on metabolic syndrome’ to ‘has been reported in metabolic syndrome’ and insert ‘in conditions including’ before ‘diabetes’ on line 140.

**Response:**

We corrected as you pointed out.

**Comment 8:**

Lines 159-160, change ‘items’ to ‘item’ and don’t need ‘strongly’.

**Response:**

We corrected as you pointed out.

**Comment 9:**

Line 162, change ‘ASD conducted an intelligence test’ to ‘an intelligence test was conducted in the ASD participants’.

**Response:**

We corrected as you pointed out.

**Comment 10:**

Results- Table 3 (line 286) change ‘intellectual functions of ASD’ to ‘intellectual function among ASD participants’.

**Response:**

We corrected as you pointed out.

**Comment 11:**

Discussion- at the end- still over stated- this finding is FAR too premature to make the recommendation that this is ‘an objective easily measured indicator that could be used in clinical practice to assess stress and clinical state’. It may certainly be worthy of further exploration, but this study is not sufficient to make such a clinical practice recommendation at this time.

**Response:**

We appreciate the Reviewer's comment on this point.

As you pointed out, the results of this study do not currently have any solid evidence to use in clinical practice. Therefore, we changed "suggest" to "may be" to soften the expression.

Again, thank you for giving us the opportunity to strengthen our manuscript with your valuable comments and queries. We have worked hard to incorporate your feedback and hope that these revisions persuade you to accept our submission.

Masahito Morimoto (Corresponding Author)

Japanese Red Cross Tokushima Hinomine Rehabilitation Center for People with Disabilities

Address: 4-1Shinbiraki, chuden-cho, Komathushima-shi, Tokushima, 773-0015 Japan

Phone : +81-885-32-0903	Fax : +81-885-33-3037

E-mail address: [morimoto@hinomine-mrc.jp](mailto:morimoto@hinomine-mrc.jp)
